# Analysis of the Effect of Slow-Varying Errors on Rotary Modulation Systems

**DOI:** 10.3390/s24155025

**Published:** 2024-08-03

**Authors:** Yabo Wang, Sizhuo Chen, Heng Li, Yueyang Ben

**Affiliations:** 1 Wuhan Second Ship Research and Design Institute, Wuhan 430205, China; 18007195551m@sina.cn; 2 School of Intelligent Science and Engineer, Harbin Engineering University, Harbin 150001, China; 18045130386@163.com (H.L.); byy@hrbeu.edu.cn (Y.B.)

**Keywords:** rotational modulation, slow-varying errors, continuous rotation, rotation–stop combination

## Abstract

Rotation modulation is a technique that relies on the specific rotation of an inertial measurement unit (IMU) to achieve the self-compensation of device errors. Common rotation schemes are classified into two modes: continuous rotation and a rotation–stop combination. Aiming at the problem of the poor modulation of slow-varying errors in the rotation–stop combination mode, a detailed analysis is conducted on the modulation effects of slow-varying errors in three schemes employing different rotation modes. Firstly, a detailed mathematical analysis is performed on the influence of gyro slow-varying drifts on two rotation modes, and the analysis results are validated through simulations. Subsequently, simulation experiments are conducted on three schemes to analyze their modulation effects on the slow-varying errors of inertial devices. The simulation results reveal that the modified dual-axis rotation scheme exhibits superior modulation effects on the slow-varying errors of inertial devices compared to the dual-axis sixteen-position rotation scheme and the multi-axis alternating continuous rotation scheme.

## 1. Introduction

Inertial navigation systems’ accuracy is primarily impacted by errors in inertial devices. Achieving substantial improvements in device precision within a short timeframe is challenging, mainly due to limitations in manufacturing expertise and economic factors. Rotation modulation employs the specific rotation of inertial devices to achieve self-compensation for errors. Within a single rotation cycle, it effectively corrects constant drifts, scale factor errors, and installation discrepancies. Thus, rotation modulation technology serves as an effective method to enhance navigation positioning accuracy using existing inertial devices.

Rotational inertial navigation systems (RINSs) can be classified into different types, including single-axis [1,2], dual-axis [3], and tri-axis systems [4]. These systems employ different rotation modes, including continuous rotation [5] and rotation–stop combination [6,7].

Regardless of the rotation mode used in the rotation scheme, the single-axis rotation scheme can only modulate constant and slow-varying errors that are perpendicular to the rotation axis, and it is unable to modulate errors along the rotation axis [8,9]. The dual-axis multi-position rotation scheme can modulate the constant drifts of three gyroscopes to zero [10]. It mostly adopts the rotation–stop combination mode, using the sixteen-position rotation scheme [11]. By maintaining stability at symmetrical positions, it effectively compensates for the IMU constant errors in the multi-position rotation scheme. Building upon the dual-axis sixteen-position rotation scheme, the dual-axis multi-position rotation scheme has further evolved to include the thirty-two-position [12] and the sixty-four-position [13] rotation schemes.

The existing literature primarily addresses the constant errors of inertial devices within rotation schemes, often neglecting the slow-varying errors arising from environmental and temperature variations. Should gyro and accelerometer errors exhibit gradual changes over time, the modulation efficacy of the dual-axis multi-position rotation scheme would diminish. In contrast, continuous rotation emerges as a superior and more effective method for mitigating slow-varying errors [14]. 

In order to mitigate slow-varying errors and enhance navigation accuracy, improvements to the rotation scheme are crucial. Lu, Y., et al. proposed a multi-axis alternating continuous rotation scheme [15], which, under similar conditions, enhances navigation accuracy by 28% compared to the single-axis continuous rotation scheme. Li, Q., et al. combined the single-axis continuous rotation scheme with the eight-position rotation scheme to propose a modified dual-axis rotation scheme [16], significantly improving navigation accuracy compared to the conventional eight-position rotation scheme. The rotation modes utilized in the aforementioned schemes encompass continuous rotation and the rotation–stop combination mode. Although both modes demonstrate some level of modulation on slow-varying errors, a comprehensive elucidation of their impact on the RINS navigation precision is still lacking.

The article begins with a mathematical analysis of the impact of gyro slow-varying drifts on the continuous rotation mode and rotation–stop combination mode, followed by a validation of the analysis results through simulations. Subsequently, simulation experiments are carried out on the three schemes mentioned in the article: the dual-axis sixteen-position rotation scheme, the multi-axis alternating continuous rotation scheme, and the modified dual-axis rotation scheme. The aim is to analyze the impact of slow-varying errors on navigation accuracy. The simulation results indicate that the modified dual-axis rotation scheme demonstrates superior modulation effects on the slow-varying errors of inertial devices compared to both the dual-axis sixteen-position rotation scheme and the multi-axis alternating continuous rotation scheme.

The other sections are organized as follows: Section 2 defines coordinate systems, outlines the basic principles of rotation modulation, and introduces three rotation schemes. Section 3 analyzes the impact of slow-varying errors on rotation modes. Section 4 presents simulation experiments. Finally, conclusions are drawn in Section 5.

## 2. Introduction of RINS

**Figure 1:**
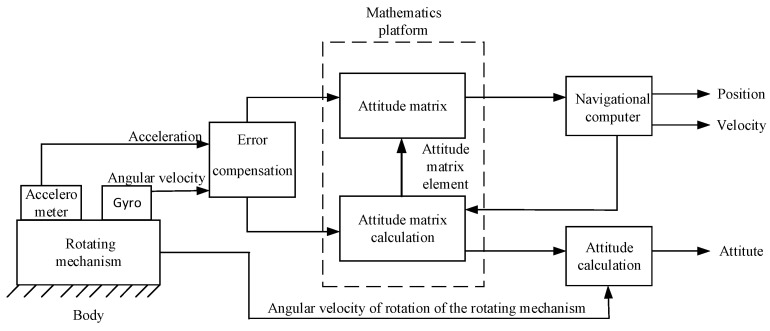
The structure diagram of the rotational strap-down inertial navigation system.

The rotational strap-down inertial navigation system is equivalent to mounting the strap-down inertial navigation system on a turntable with a rotation mechanism. The navigation solution also employs the strap-down inertial navigation algorithm. Since the IMU rotates relative to the carrier, the attitude information directly calculated by navigation only represents the IMU’s attitude. To obtain the carrier’s attitude information, it is necessary to add the rotational angle of the IMU relative to the carrier. The structure diagram of the rotational strap-down inertial navigation system is shown in Figure 1.

### 2.1. Definition of Coordinate System

The body coordinate system (b-system) is defined as the up, right, and front directions of the RINS. The navigation coordinate system (n-system) is defined as the east, north, and up directions of the current position. The IMU coordinate system (s-system) is an orthogonal coordinate system used for the gyro and accelerometer data output. The s-system and b-system are perfectly aligned when the IMU rotation angle is zero.

### 2.2. Rotary Modulation Fundamentals

To facilitate the analysis, we assume that at the initial moment, the b-system coincides with the n-system, and the s-system coincides with the b-system. The IMU then begins to rotate continuously around the ${\mathrm{oz}}_{s}$-axis with a constant angular velocity. A counterclockwise rotation is considered positive, while a clockwise rotation is considered negative. The direction cosine matrix between the s-system and the b-system at time t is as follows:

(1) $$C_{s}^{b}={\left(C_{b}^{s}\right)}^{T}=\left[\begin{array}{ccc} \cos\omega t & -\sin\omega t & 0\\ \sin\omega t & \cos\omega t & 0\\ 0 & 0 & 1 \end{array}\right]$$

where “T” is the transpose of the matrix.

**Figure 2:**
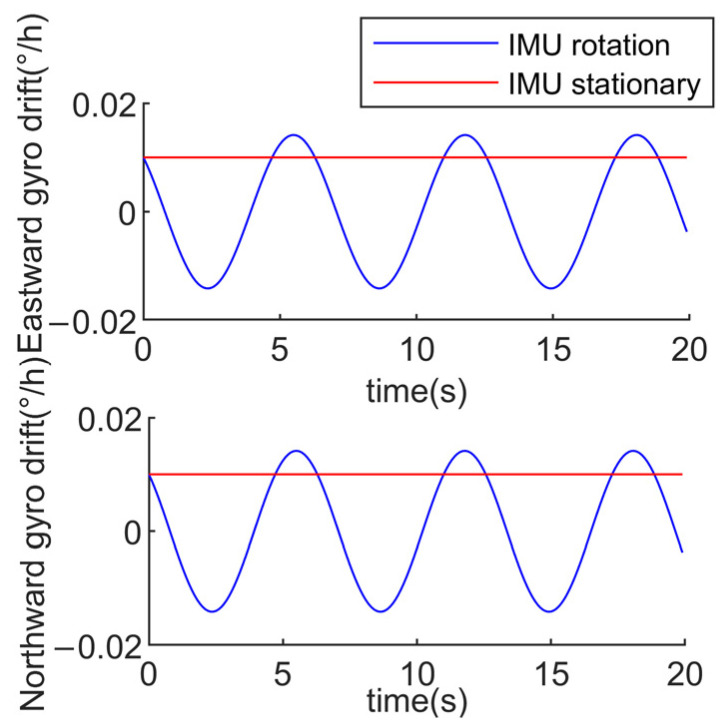
Equivalent gyro drift in the n-system.

Since the calculation process of the inertial navigation system is carried out under the n-system, in order to analyze the problem in a simple and intuitive manner, this chapter assumes that the b-system coincides with the n-system, i.e., $C_{s}^{n}=C_{b}^{n}C_{s}^{b}=C_{s}^{b}$. Then, the modulation results of the inertial device errors in the n-system at time t can be expressed as follows:

(2) $$\left[\begin{array}{c} \varepsilon_{E}\\ \varepsilon_{N}\\ \varepsilon_{U} \end{array}\right]=C_{s}^{n}\left[\begin{array}{c} \varepsilon_{x}^{s}\\ \varepsilon_{y}^{s}\\ \varepsilon_{z}^{s} \end{array}\right]=\left[\begin{array}{c} \varepsilon_{x}^{s}\cos\omega t-\varepsilon_{y}^{s}\sin\omega t\\ \varepsilon_{x}^{s}\sin\omega t+\varepsilon_{y}^{s}\cos\omega t\\ \varepsilon_{z}^{s} \end{array}\right]$$

(3) $$\left[\begin{array}{c} \nabla_{E}\\ \nabla_{N}\\ \nabla_{U} \end{array}\right]=C_{s}^{n}\left[\begin{array}{c} \nabla_{x}^{s}\\ \nabla_{y}^{s}\\ \nabla_{z}^{s} \end{array}\right]=\left[\begin{array}{c} \nabla_{x}^{s}\cos\omega t-\nabla_{y}^{s}\sin\omega t\\ \nabla_{x}^{s}\sin\omega t+\nabla_{y}^{s}\cos\omega t\\ \nabla_{z}^{s} \end{array}\right]$$

where $\varepsilon_{x}^{s}$, $\varepsilon_{y}^{s}$, $\varepsilon_{z}^{s}$ are the gyro constant value drifts; $\nabla_{x}^{s}$, $\nabla_{y}^{s}$, $\nabla_{z}^{s}$ are the accelerometer biases; $\varepsilon_{E}$, $\varepsilon_{N}$, $\varepsilon_{U}$ are the modulated eastward, northward, and skyward equivalent gyro drifts; and $\nabla_{E}$, $\nabla_{N}$, $\nabla_{U}$ are the modulated eastward, northward, and skyward equivalent accelerometer biases. To visually demonstrate the modulation effect of rotation modulation on the gyro constant drift and accelerometer bias, we conduct a simple numerical calculation. Assuming $\varepsilon_{x}^{s}=\varepsilon_{y}^{s}=0.01^{{}^{\circ}}/h$, the equivalent gyro drift in the navigation system, both in the case of IMU rotation and in the absence of rotation, is shown in Figure 2.

From Figure 2, it is evident that the errors in the horizontal direction of the inertial devices, caused by IMU rotation around the ${\mathrm{oz}}_{s}$-axis, are modulated into a zero-mean periodic form. Therefore, rotation around the ${\mathrm{oz}}_{s}$-axis does not affect navigation accuracy. However, there is no modulation of errors along the rotation axis in the inertial devices. Consequently, single-axis rotation modulation technology can only modulate the constant errors of inertial devices perpendicular to the rotation axis direction. Errors along the rotation axis still propagate navigation errors in the original manner. Thus, to effectively modulate errors in all three directions of the inertial devices, at least dual-axis rotation is required.

### 2.3. Rotation Schemes

**Figure 3:**
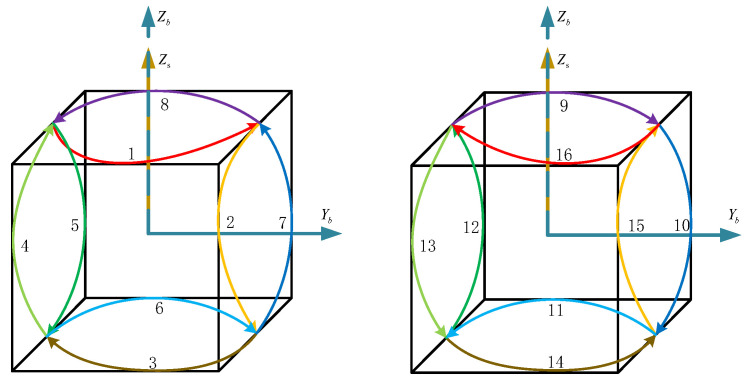
The dual-axis sixteen-position rotation scheme.

The dual-axis sixteen-position rotation scheme [11] primarily adopts a rotation–stop combination mode, leveraging error cancelation at symmetric positions to enhance navigation accuracy. The illustration of the dual-axis sixteen-position rotation scheme is depicted in Figure 3. Figure 3 illustrates the dual-axis sixteen-position rotation scheme.

**Figure 4:**
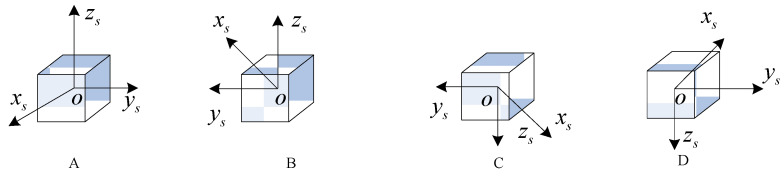
(**A**) First position of IMU. (**B**) Second position of IMU. (**C**) Third position of IMU. (**D**) Fourth position of IMU [16].

In the dual-axis sixteen-position rotation scheme, the IMU rotates to a specific position, remains there for a period, and then rotates to the next position. As shown in Figure 4, when the IMU rotates to position A, it stays there for a while before rotating to position B and pausing again. At positions A and B, the directions of the $x_{s}$-axis and $y_{s}$-axis are opposite, thus canceling out the effects of x-axis and y-axis gyro drift on the navigation results through symmetric positions. However, gyro drifts also include errors with slow variations. Assuming the initial gyro drift is 0.1°/h and the gyro drift changes over time according to the function y = 0.001t + 0.1, the gyro drift at position B will differ from that at position A, preventing complete symmetrical cancelation and leading to a decrease in navigation accuracy. Additionally, in the sixteen-position rotation scheme, one modulation cycle includes sixteen rotation periods and sixteen stop periods. Assuming the rotation time for the dual-axis scheme is 30 s and the stop time is 30 s, while the rotation time for the single-axis continuous rotation scheme is 60 s, the modulation cycle for the dual-axis scheme is 12 min, whereas the modulation cycle for the single-axis continuous rotation is only 2 min. Therefore, the rotational modulation effect on slow-varying drift in the dual-axis sixteen-position rotation scheme is limited.

**Figure 5:**
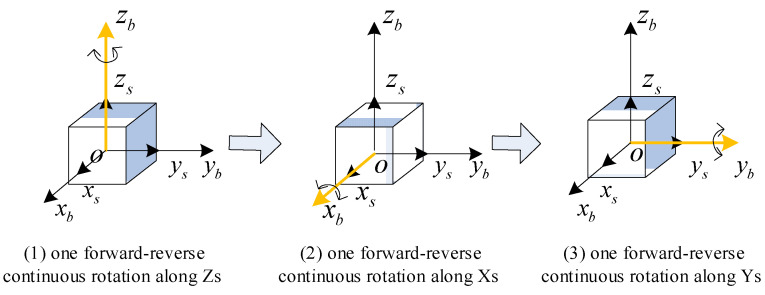
The multi-axis alternating continuous rotation scheme.

The multi-axis alternating continuous rotation scheme [15] is shown in Figure 5. After the system enters the navigation state, the IMU rotates positively and negatively around the $z_{b}$, $x_{b}$, and $y_{b}$ axes in sequence. Compared to the sixteen-position rotation scheme, the multi-axis alternating continuous rotation scheme can mitigate the impact of slow-varying errors without causing significant velocity error oscillations.

Most single-axis rotation schemes adopt a continuous rotation mode, while most dual-axis multi-position rotation schemes adopt a rotation–stop combination mode. In the rotation–stop combination mode, the RINS primarily relies on the periodic reversal of sensitive axes to place the gyro at symmetric positive and negative positions, thereby canceling out the effects of gyro drift. In the rotation–stop combination mode, the modulation period of gyro drift is typically longer than that in continuous rotation mode. Therefore, the modulation effect of slow-varying errors in inertial devices may be limited. Therefore, this article references a modified dual-axis rotation scheme [16] designed by Li, Q., et al., which combines continuous rotation mode with the rotation–stop combination mode. The modified scheme significantly reduces the time required to modulate gyro horizontal drifts to zero mean and enhances the modulation effect of slow-varying gyro drifts.

**Figure 6:**
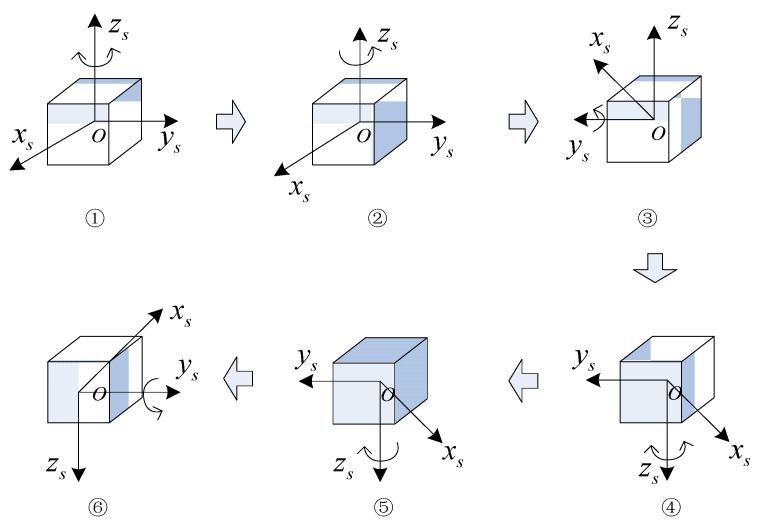
Stage I of the proposed dual-axis rotation scheme.

The rotational mode of the first phase of the rotation scheme is illustrated in Figure 6, with the specific process outlined below:

(1) The IMU is rotated clockwise and counterclockwise about the $z_{s}$-axis n times, respectively, n being a positive integer.
(2) The IMU is rotated 180° positively about the $z_{s}$-axis.
(3) The IMU is rotated 180° negatively about the $y_{s}$-axis.
(4) The IMU is rotated clockwise and counterclockwise around the $z_{s}$-axis n times, respectively.
(5) The IMU is rotated 180° positively downward around the $z_{s}$-axis.
(6) The IMU is positively rotated 180° about the $y_{s}$-axis.

**Figure 7:**
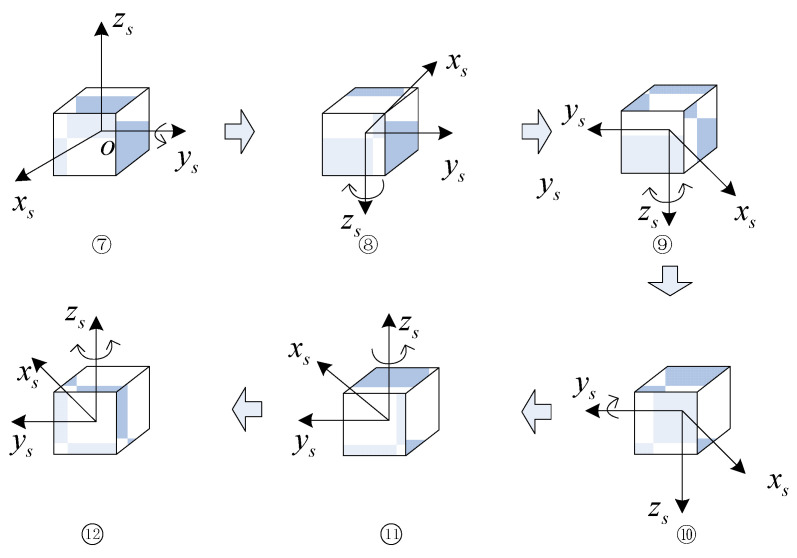
Stage II of the proposed dual-axis rotation scheme.

In the second stage, the position sequence of the IMU is the reverse sequence of the first stage, and the semicircular rotations along the z-axis and along the $y_{s}$-axis can form a whole cycle with the first stage, so the rotation of the second stage is shown in Figure 7.

## 3. Slow-Varying Errors Modulation Effect Analysis

The main difference among the three schemes mentioned above lies in the type of rotation mode used: continuous rotation or rotation–stop combination. To analyze the modulation effect of the slow-varying errors in these schemes, we first analyze the impact of gyro’s slow-varying drifts on both the continuous rotation mode and the rotation–stop combination mode.

From Equations (2) and (3), it is evident that single-axis rotation modulation can completely modulate the constant errors of the gyro in the horizontal direction. However, during the operational process, changes in the environment, parameters, and other factors lead to significant variations in gyro drifts, scale factors, and the orientation of gyro sensitive axes with changes in temperature or time [14]. These variations consequently affect the effectiveness of rotation modulation. Therefore, in order to clearly contrast the impact of gyro slow-varying drifts on the continuous rotation and rotation–stop combination modes, two x-axis gyro slow-varying drifts models are established. One model represents gyro drifts as a function of time, while the other models it as a first-order Markov process. Through comparative experiments, we deduced the effect of gyro slow-varying drifts on these two rotation modes.

### 3.1. Exponential Gyro Drifts

Assuming that the x-axis gyro drift varies with time t, the relationship is given by [15]:

(4) $$\Delta \varepsilon^{s}=\Delta \varepsilon^{s}\cdot e^{-0.001t}$$

From Equation (4), the projection of the x-axis gyro drift onto the $x_{b}$-axis is:

(5) $$\Delta \varepsilon_{xb}^{s}=\Delta \varepsilon_{x}^{s}\cdot e^{-0.001t}\cos\omega t$$

From Equation (5), we can see that the presence of gyro slow-varying drifts affects the modulation effect of rotation, thereby impacting navigation accuracy. To visually compare the impact of gyro slow-varying drifts on the modulation effect of the two rotation schemes, we will now conduct simulation experiments.

**Figure 8:**
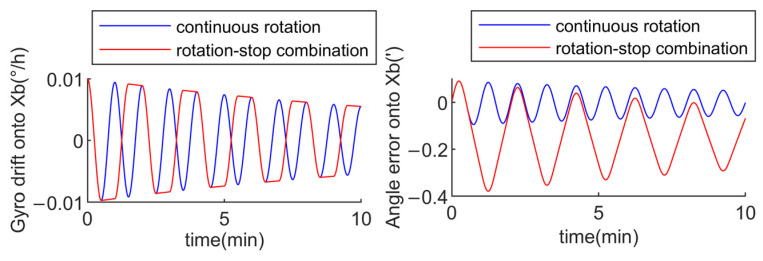
Modulation effect of continuous rotation mode and rotation-stop combination mode on gyro drift.

Assuming the x-axis gyro drift is $0.01^{{}^{\circ}}/h$, and the rotation angular velocity for both the continuous rotation mode and the rotation–stop mode is $6^{{}^{\circ}}/s$, the rotation cycle for the continuous rotation mode is 120 s, with a specific sequence of 60 s clockwise rotation followed by 60 s counterclockwise rotation. For the rotation–stop combination mode, the rotation period is 240 s, with a sequence of 30 s clockwise rotation, 30 s stop, 30 s clockwise rotation, 30 s stop, 30 s counterclockwise rotation, 30 s stop, 30 s counterclockwise rotation, and 30 s stop. Through simulation, the $x_{b}$-axis gyro drift and $x_{b}$-axis angle error for both schemes are obtained within 10 min as follows:

From Figure 8, it is evident that the $x_{b}$-axis angle error of the continuous rotation mode is modulated into a periodic form, with a mean value of $-0.0034^{\prime \prime }$ and an amplitude of $-0.095^{\prime \prime }$. Similarly, the $x_{b}$-axis angle error of the rotation–stop combination mode is also modulated into a periodic form. However, the $x_{b}$-axis angle error in the rotation–stop combination mode is significantly larger than that in the continuous rotation mode, with a mean value of $-0.1493^{\prime \prime }$ and an amplitude of $-0.379^{\prime \prime }$. By comparing the two, it can be concluded that the continuous rotation mode exhibits a smaller amplitude and mean value for angle error, indicating a better modulation effect on gyro slow-varying drifts.

### 3.2. Markovian Gyro Drifts

In order to further analyze the modulation effect of the slow-varying errors by two rotation modes, we will establish the gyro drifts as a first-order Markov model.

(6) $$\varepsilon_{r+1}^{s}=e^{-\alpha T}\varepsilon_{r}^{s}+w_{r}$$

In the equation, α is the anti-correlation time constant, T is the time interval, and $w_{r}$ is zero-mean Gaussian white noise.

From Equation (6), the projection of the x-axis gyro drift onto the $x_{b}$-axis is:

(7) $$\Delta \varepsilon_{xb}^{s}=\Delta \varepsilon_{r}^{s}\cdot \cos\omega t$$

From Equation (7), it is evident that the presence of gyro slow-varying drifts leads to a decrease in the modulation effect of single-axis rotation modulation on the horizontal axis. To more intuitively compare the impact of gyro slow-varying drifts on the continuous rotation mode and rotation–stop combination mode, we will conduct simulation experiments.

**Figure 9:**
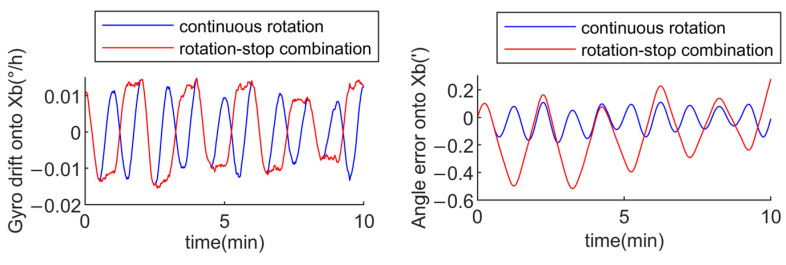
Modulation effect of continuous rotation mode and rotation-stop combination mode on gyro drift.

Assuming the x-axis gyro drift is $0.01^{{}^{\circ}}/h$ and the anti-correlation time constant is $\alpha =1/\left(7200s\right)$, the rotation cycle for both the continuous rotation mode and the rotation–stop mode is the same as mentioned earlier. Through simulation, the $x_{b}$-axis gyro drift and $x_{b}$-axis angle error for both schemes are obtained within 10 min as follows:

From Figure 9, it is evident that modeling the gyro drift as a first-order Markov process has a smaller impact on the $x_{b}$-axis angle error in the continuous rotation mode. The mean value of the modulated $x_{b}$-axis angle error is $-0.0188^{\prime \prime }$, with an amplitude of $-0.167^{\prime \prime }$. Compared to the continuous rotation mode, the gyro error with a first-order Markov process has a larger impact on the $x_{b}$-axis angle error in the rotation–stop combination mode. The mean value of the $x_{b}$-axis angle error is $-0.1213^{\prime \prime }$, with an amplitude of $-0.525^{\prime \prime }$. By comparing the two modes, it can be concluded that the continuous rotation mode has a smaller amplitude and mean value for the x-axis angle error, indicating a better modulation effect on the gyro slow-varying drifts.

According to Table 1, gyro drifts with both time-varying functions and a first-order Markov process have a lesser impact on the modulation effect of the continuous rotation mode compared to the rotation–stop combination mode. This indicates that the continuous rotation mode is better at modulating the gyro slow-varying drifts, making it the preferred rotation mode to consider during the design of rotation schemes.

## 4. Simulation

The preceding analysis explored how gyro slow-varying drifts affect the continuous rotation mode and the rotation–stop combination mode. To further investigate the efficacy of the three mentioned schemes in modulating the slow-varying errors of inertial devices, simulations are carried out under identical conditions for the dual-axis sixteen-position rotation scheme, the multi-axis alternating continuous rotation scheme, and the modified dual-axis rotation scheme.

In the simulation, the initial position is set at 125° E, 45° N, with a navigation time of 72 h. Parameter Settings are shown in Table 2. The gyro drifts are $0.0065^{{}^{\circ}}/h$, and the accelerometer biases are 20 μg. Both the gyro drifts and accelerometer biases are modeled as first-order Markov processes with anti-correlation time constants of $\alpha =1/\left(7200s\right)$.

The positions of the IMU for the dual-axis sixteen-position rotation scheme, the multi-axis alternating continuous rotation scheme, and the modified dual-axis rotation scheme are described in Section 2.3. In the dual-axis sixteen-position rotation scheme, the IMU stays at each position for 30 s. The rotational speed for all three rotation modulation schemes mentioned above is $6^{{}^{\circ}}/s$.

Based on the analysis in Section 3.2, it is concluded that the continuous rotation mode can better modulate gyro slow-varying drifts. To further verify this analysis, the paper conducts static simulations with the addition of gyro drifts and accelerometer biases, modeled as first-order Markov processes.

Under the above simulation conditions, velocity errors are obtained, including the eastward velocity error ($\Delta V_{E}$), the northward velocity error ($\Delta V_{N}$), and the position errors, including the latitude error and the longitude error.

**Figure 10:**
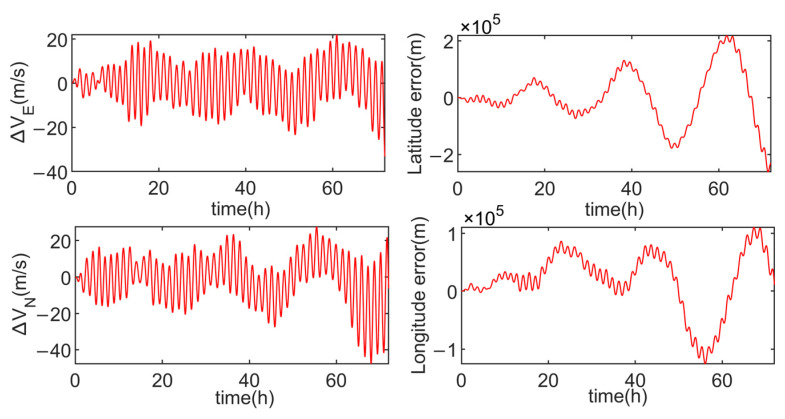
Velocity errors and position errors in the stationary state.

Firstly, simulation experiments are conducted on the inertial navigation system in a stationary state, and the results are shown in Figure 10.

**Figure 11:**
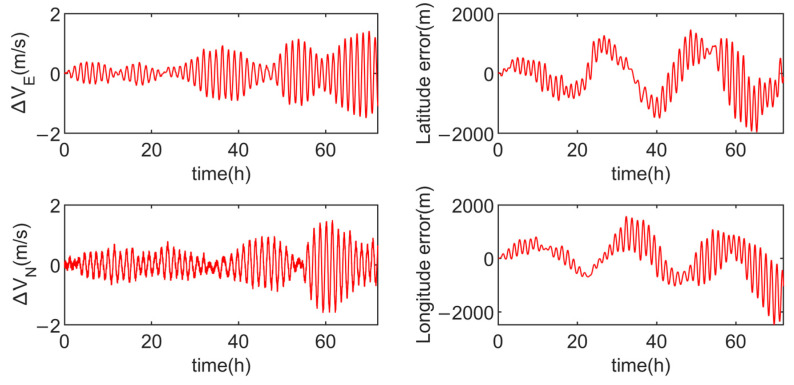
The velocity errors and positioning errors of the dual-axis sixteen-position rotation scheme.

After conducting simulation experiments on the dual-axis sixteen-position rotation scheme, the results are shown in Figure 11.

**Figure 12:**
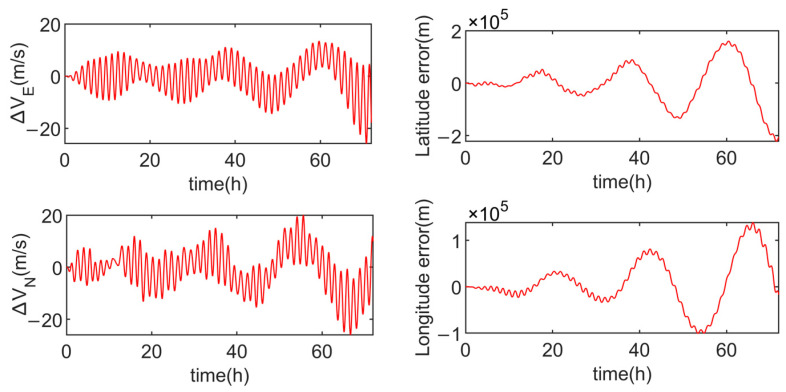
Velocity errors and position errors of the multi-axis alternating continuous rotation scheme.

**Figure 13:**
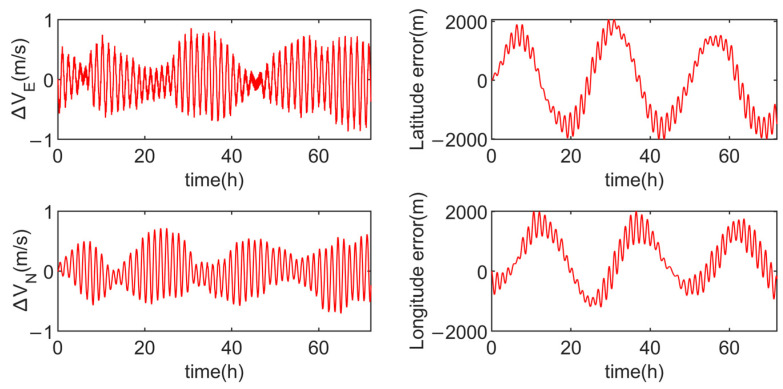
Velocity errors and position errors of the modified dual-axis rotation scheme.

Simulation experiments are conducted for the multi-axis alternating continuous rotation scheme and the modified dual-axis rotation scheme, with the results shown in Figure 12 and Figure 13.

In order to intuitively demonstrate the advantages and disadvantages of the three schemes, Table 3 compares the velocity errors of each scheme. From Table 3, it is evident that the multi-axis alternating continuous rotation scheme has a relatively poor modulation effect on the inertial device errors, resulting in larger amplitudes in both velocity and position errors. The modified dual-axis rotation scheme, on the other hand, exhibits the smallest amplitudes in the eastward velocity error and the northward velocity error, with position error amplitudes similar to the dual-axis sixteen-position scheme.

As observed in Figure 11 and Figure 13, due to the presence of slow-varying errors, the velocity and position error amplitudes of the dual-axis sixteen-position rotation scheme increase over time. In contrast, the modified dual-axis rotation scheme does not exhibit this issue. This is attributed to its rotational mode, which combines continuous rotation with rotation–stop periods, leading to shorter rotation cycles and a superior modulation effect on slow-varying errors.

The simulation results indicate that, compared to the multi-axis alternating continuous rotation scheme, the modified dual-axis rotation scheme effectively mitigates the impact of inertial device errors on navigation accuracy. Furthermore, compared to the dual-axis sixteen-position scheme, the modified dual-axis rotation scheme is able to modulate slow-varying errors, thereby enhancing navigation accuracy during long-duration navigation.

## 5. Discussion

The dual-axis rotation scheme primarily adopts a rotation–stop combination mode. However, due to significant variations in the inertial device errors with temperature or time changes, continuous rotation is more effective at offsetting IMU-related errors than maintaining stability at symmetric positions. The analysis of the slow-varying errors’ modulation indicates that continuous rotation mode is superior at modulating slow-varying errors compared to the rotation–stop combination mode. The simulation experiments demonstrate that the modified dual-axis rotation scheme is more adept at modulating gyro slow-varying errors than both the multi-axis alternating continuous rotation scheme and the dual-axis sixteen-position rotation scheme. This indicates that combining the continuous rotation mode with the rotation–stop mode can modulate drift in all three axes to zero mean and suppress slow-varying errors. Although this study only simulated one type of rotational scheme, combining continuous rotation with rotation–stop mode, it still has certain limitations. Nevertheless, the research results are significant for further enhancing navigation capabilities.

## Figures and Tables

**Table 1:** Gyro angle error.

	The Mean in Section 3.1	The Amplitude in Section 3.1	The Mean in Section 3.2	The Amplitude in Section 3.2
The continuous rotation mode	$-0.003^{\prime \prime }$	$-0.095^{\prime \prime }$	$-0.019^{\prime \prime }$	$-0.183^{\prime \prime }$
The rotation–stop mode	$-0.149^{\prime \prime }$	$-0.379^{\prime \prime }$	$-0.121^{\prime \prime }$	$-0.517^{\prime \prime }$

**Table 2:** Setting of error parameters for simulations.

Error Paramrters	Value
Constant gyro drifts	$0.0065^{{}^{\circ}}h^{-1}$
Constant accelerometer biases	20 μg

**Table 3:** Amplitude of velocity error and position error for the three schemes.

	Eastward Velocity Error	Northward Velocity Error	Latitude Error	Longitude Error
The stationary state	33.2 m/s	47.7 m/s	$2.6\times 10^{5}\;m$	$1.3\times 10^{5}\;m$
The dual-axis sixteen-position rotation scheme	1.5 m/s	1.6 m/s	1963.5 m	2487.3 m
The multi-axis alternating continuous rotation scheme	25.9 m/s	25.7 m/s	$2.2\times 10^{5}\;m$	$1.4\times 10^{5}\;m$
The modified dual-axis rotation scheme	0.87 m/s	0.71 m/s	2057.9 m	2013.4 m

## Data Availability

The data that support the findings of this study are available from the corresponding author upon reasonable request.
